# The choice between surgical scrubbing and sterile covering before or after induction of anaesthesia: A prospective study

**DOI:** 10.12688/f1000research.11965.2

**Published:** 2017-08-09

**Authors:** Irene Sellbrandt, Metha Brattwall, Pether Jildenstål, Margareta Warrén Stomberg, Jan Jakobsson

**Affiliations:** 1 Department of Anaesthesia, Capio Lundby Hospital, Gothenburg, 417 17, Sweden; 2 Department of Anaesthesia and Intensive Care, Sahlgrenska University Hospital, Mölndal, 413 45, Sweden; 3 Department of Anaesthesiology and Intensive Care Medicine, Institute of Health and Care Sciences, The Sahlgrenska Academy, Gothenburg University, Gothenburg, 413 46, Sweden; 4 Department of Anaesthesia, Institution for Clinical Science, Karolinska Institutet at Danderyds University Hospital, Stockholm, 182 88, Sweden

**Keywords:** Day surgery, operating theatre turn around, theatre efficacy, surgical scrubbing, sterile covering

## Abstract

***Background***: Day surgery is increasing, and safe and effective logistics are sought. One part of the in-theatre logistics commonly discussed is whether surgical scrub and sterile covering should be done before or after induction of anaesthesia. The aim of the present study was to compare the impact of surgical scrub and sterile covering before vs. after the induction of anaesthesia in male patients scheduled for open hernia repair.

***Methods***: This is a prospective randomised study. Sixty ASA 1-3 patients scheduled for open hernia repair were randomised to surgical scrub and sterile covering before or after induction of anaesthesia; group “awake” and “anaesthetised”. Need for vasoactive medication during anaesthesia was primary study objective. Duration of anaesthesia and surgery, theatre time, recovery room stay and time to discharge, patients and theatre nurses experiences and willingness to have the same logistics on further potential surgeries, by a questionnaire provided before discharge was also assessed.

***Results***: The duration of anaesthesia was shorter and doses of propofol and remifentanil were reduced by 10 and 13%, respectively, in the awake group. We found still no difference in the need for vasoactive medication during anaesthesia Time in recovery area was significantly reduced in the awake group 39 (SD 15) vs. 48 SD 16) (p<0.05), but time to discharge was not different. There was further no difference in the patients’ assessment of quality of care, and only one patient in the awake group would prefer to be anaesthetised on a future procedure. All nurses found pre-anaesthesia scrubbing acceptable as routine.

***Conclusion***: Surgical scrub and sterile covering before the induction of anaesthesia can be done safely and without jeopardising patients’ quality of care and possibly improve perioperative logistics. Further studies are warranted assessing impact of awake scrubbing and sterile covering on quality and efficacy of perioperative care.

## Introduction

Day surgery, where the patient leaves the hospital on the same day as surgery, is increasing. Shortening a hospital stay is associated to several benefits: Early ambulation reduces the risk for thromboembolic complications, as well as postoperative infections; and it will reduce health care costs. Thus, its implementation is of value for both patients and society. However, day surgery calls for good perioperative care, enabling rapid recovery to send patients safely home after a few hours following the end of surgery/anaesthesia. Shortening anaesthesia time, avoiding unnecessary anaesthetic exposure, has several potential benefits, including avoiding unnecessary cardiovascular depression, since there is a miss-match between anaesthetic depression and stimuli, thus requiring vasoactive support, improving early recovery, and reducing the amount of anaesthetic used.

The aim of the present study was to compare surgical scrub and sterile covering before *vs.* after induction of anaesthesia. Our hypothesis was that avoiding prolonged anaesthesia by inducing anaesthesia prior to surgical scrubbing and sterile covering would reduce the need for vasoactive medication. Additionally, the study aimed to determine if this different theatre logistic further affect drug doses of anaesthetic agents, post-anaesthesia care unit (PACU) time and quality of care.

## Methods

### Ethical approval

The study protocol has been reviewed and approved by the Gothenburg Ethical Committee (Dnr. 751-16 scientific secretary Sven Wallerstedt, October 24 ^th^ 2016).

### Study group

The study was conducted at Capio Lundby Hospital in Gothenburg, November 2016 – February 2017. Male patients scheduled for elective open hernia repair with a modified Lichtenstein technique under general anaesthesia were requested to participate. Exclusion criteria was severe cardiovascular, respiratory, hepatic or renal disease, and American Society of Anaesthesiology (ASA) score of >3. Sixty ASA 1–3 patients scheduled for elective open hernia repair, modified Lichtenstein procedure, participated in the study following verbal and written informed consent. These patients were randomised by envelope technique into two groups:

1. Awake group: Surgical scrub and sterile covering before induction of anaesthesia, having the patient awake but sedated.
2. Anesthetised group: Surgical scrub and sterile covering when the patient is asleep, anaesthesia induction and securing airway and start of maintenance has been initiated.

Patients received all medication and care in accordance to routine procedures of the department, apart from the scrubbing and sterile covering. Premedication was with paracetamol and diclofenac.

Anaesthesia was induced and maintained with propofol and remifentanil (total intravenous anaesthesia; TIVA). Anaesthesia was adjusted per clinical signs. No EEG-based depth of anaesthesia monitor was used. Patients had local anaesthesia in the wound area during the surgery. Postoperative nausea and vomiting (PONV) prophylaxis was administered based on risk, assessed by Apfel score.

### Data collection

Patients’ assessment of their experience being awake or anaesthetised during surgical scrub and sterile covering was collected using a postoperative survey. The survey used a visual analogue scale (VAS; 0, unacceptable to 10, fully acceptable) to describe the experience, and the question ‘would you like to have the same care if you needed further surgery?’ (yes/no/I don’t know).

Perioperative observations were collected from the patient case record.

Operating room nurses (n=7) were asked whether they found the surgical scrub and covering acceptable from a patient care perspective (VAS scale 0 not at all – 10 fully acceptable) only for awake patients.

### Data analysis

All data is presented as mean ± standard deviation (SD), unless otherwise stated. Differences between groups’ continuous data, e.g. demographics and perioperative observation were assessed by Student’s *t*-test, and categorical data with Chi-square test. A p<0.05 was considered statistically significant. Data was analysed with StatView (v1.04) for MAC.

The number of patients studied is based on a power analysis from findings in a pilot study; awake surgical scrub and sterile covering should reduce the need for vasoactive medication. A difference of 10 to 5 mg composite with SD of 6 with a power of 80% would require two groups of patients with 23 each to show a difference p<0.05.

## Results

All 60 patients asked for participation accepted and signed informed consent. Assessment of quality of care during surgical scrub and sterile covering was assessed by all 60 patients; three patients were excluded from analysis of anaesthesia and recovery, as the surgery became more extensive than planned or for social reasons, and the patients were kept as inpatients. There were only minor demographic differences between the groups: the mean age of the awake group was 5 years older, but the ASA class was not different ( Table 1).

Duration of anaesthesia and time with laryngeal mask airway was shorter in the awake group ( Table 2). The amount of propofol and remifentanil required was also lower in the awake group: 10% reduction in propofol and 13% reduction of remifentanil, but this difference in drug amount was not significant. We found however no difference in vasoactive need between groups. There were no differences in early recovery or vital signs, and first pain was reported at similar times in both groups. Time in PACU was shorter for the awake patients (p<0.05), but time to discharge, pain and post-operative nausea and vomiting showed no difference between groups ( Table 2).

**Table 1.** Patient demographics of patients in the awake and anaesthetised group. Data is displayed as the mean (standard deviation), unless otherwise stated.

	Anaesthetised (n=30)	Awake/sedated (n=30)	P-value
Age (years)	58 (16)	63 (15)	0.2
BMI	26 (3)	26 (3)	0.4
ASA, 1/2/3	5/18/7	9/17/4	.3
Smoking, yes/no	5/25	3/27	0.7

In total, 27 of the awake patients would undergo surgery using the same logistics, two were indifferent and one was “negative”, while 21 of the anaesthetised patient would like to have the same logistics, and nine were indifferent. The theatre nurses rated patients being awake during surgical scrub and sterile covering as acceptable; 4out of the 7 nurses rated 10(scale 0 -10), while the remaining rated; 1 nurse made a rating of 6 and 2 nurses, both rated the process of washing and dressing as 8 (on the 0 -10 scale). All 7 nurses involved in the patient care considered it feasible to perform surgical scrub and sterile covering before induction of anaesthesia as routine procedure.

**Table 2.** Perioperative observations of the patients in the awake and anaesthetised group. Data is displayed as the mean (standard deviation), unless otherwise stated. Two patients in the awake group and one in the anaesthetised group were excluded from analysis, since they were kept as inpatients. Surgery time is defined as the time the patient is being operated on; theatre time is defined as the entrance to theatre to leaving for PACU. LMA, laryngeal mask airway; PACU, post-anaesthesia care unit; VAS, visual analog scale.

	Anaesthetised (n=29)	Awake/sedated (n=28)	P-value
Anaesthesia (min.)	64 (10)	60 (14)	0.17
Propofol (mg)	506 (114)	455 (119)	0.10
Remifentanil (µg)	837 (205)	731 (256)	0.08
Ephedrine (mg), median (range)	0 (0-15)	0 (0-25)	0.16
Phenylephrine (mg), median (range)	0 (0-0.8)	0 (0-0.4)	0.46
Time with LMA (min.)	58 (10)	52 (14)	0.068
Surgery (min.)	39 (9)	38 (13)	0.78
Theatre (min.)	70 (10)	69 (13)	0.77
PACU (min)	48 (16)	39 (15)	p0.042
Max pain during PACU (VAS)	1.8 (2.7)	1.2 (2.1)	0.33
Time to discharge (min.)	224 (58)	235 (65)	0.45
Patient assessed quality of care (VAS), median (range)	10 (6-10)	10 (3-10)	0.09

Demographics, perioperative observations, and response to questionnaire of the patients undergoing surgical scrub and covering pre and post-anaesthesia Click here for additional data file. Copyright: © 2017 Sellbrandt I et al. 2017 Data associated with the article are available under the terms of the Creative Commons Zero "No rights reserved" data waiver (CC0 1.0 Public domain dedication).

## Discussion

We could not prove our hypothesis that sterile washing and dressing before induction of anaesthesia reduced anaesthetic drug usage. But, no difference in need for vasoactive medication was found. We found however that surgical scrub and sterile covering prior to induction of anaesthesia feasible and with a maintained quality of care. We have the experience that theatre nurses in Sweden prefer the patient being asleep while surgical scrubbing and sterile covering is performed is performed. The argument is that it may be distressful for patients if they are awake during preparation, surgical scrubbing and sterile covering. However, in this study, generally patients did not mind being awake, on the contrary some patients gave positive feedback about being awake during preparation. Some nurses also feel that the liquid used for scrubbing may cause a freezing sensation in patients; we did not hear any comments supporting this notion. There are also discussions regarding that awake patients may be at an increased risk for surgical site infections (SSI). In a majority of SSI cases, the pathogen source is the native flora of the patient’s skin and there is no firm evidence that the anaesthetic technique used, i.e. patient being awake or asleep during scrub and sterile covering, should impact the infection risk ^1, 2^. Two recent studies in a huge number of patients undergoing orthopaedic procedures did not show any difference between general anaesthesia, spinal anaesthesia and peripheral blocks ^3, 4^.

Washing and dressing after induction prolongs the time of anaesthesia and to titrate optimal depth keeping patient asleep with only merely minor stimulation following induction may cause additional need for vasoactive medications and may prolong recovery. Shortening the duration of anaesthesia and drug doses may be of value, especially for elderly patients. There are studies suggesting that tailoring anaesthesia reduces the risks for cognitive side effects ^5^. We did not follow patients beyond discharge. All our patients had total intravenous anaesthesia; whether use of inhaled anaesthesia for maintenance could further improve emergence, the early recovery, and quality of recovery cannot be assessed from the present study. We found in a previous study that inhaled anaesthesia facilitates early recovery ^6^. We could however not see any reduced need for vasoactive medication, thus our primary hypothesis was negative. We cannot give any firm explanation as to why this occurred, since in the awake group the need for both propofol and remifentanil was reduced.

Theatre turnaround time is of increasing importance. Efforts to improve efficacy has been addressed in several studies. Koenig *et al.* studied anaesthesia induction when the surgeon was in theatre or not, and its impact on waiting time and unnecessary anaesthesia duration ^7^. They found a significant shortening of anaesthesia time when surgeons were readily available in the theatre. Saha *et al.* found that transfer of patient to and from theatre has a significant impact on theatre turnaround time ^8^. We found clear logistical benefits associated with the use of local anaesthesia and sedation as compared to general anaesthesia in a previous study ^9^. The benefit of local anaesthesia sedation technique has also been supported by others for vaginal prolapse surgery ^10, 11^. Open hernia repair is commonly done under local anaesthesia only ^12^. Thus, avoiding anaesthesia during surgery preparation also seems to be a feasible alternative when patients are undergoing general anaesthesia. The anaesthetised patients were somewhat younger and whether that could have impacted the results, need for vasoactive medication cannot be stated, the ASA profile was however similar between the groups.

There are limitations to our study. We studied only one procedure, elderly male patients scheduled for inguinal hernia repair. We had need for vasoactive medication as the primary study objective; this may not be the optimal choice and the number of patients studied is also relatively few. The number of patients was based on our power analysis from pilot experience. Whether these results are transferable to other procedures needs further studies. Proper information around the importance of scrubbing and covering should be given to patients, and providing light sedation should be done in accordance to a patient’s wish. Whether fine tuning anaesthetic delivery could further impact the results cannot be stated. We could not find studies assessing the surgical scrub and sterile covering impact on quality of care or theatre time events, thus we are not able to truly put our findings into perspective of previous similar results. We still believe that our findings can be of interest and importance as lean operating theatre planning is of growing importance. Further studies are indeed warranted assessing impact of awake scrubbing and sterile covering on quality and efficacy of perioperative care. There are studies looking at different anaesthetic techniques and the use of a holding area for theatre preparation, which show benefits to introducing peripheral blocks before entry to the theatre ^13^.

In conclusion, preparation, surgical scrub and sterile covering, before induction of anaesthesia is feasible, and does not jeopardise quality of care. In addition, it reduces anaesthetic agents need and may thus shorten recovery room stay.

## Data availability

The data referenced by this article are under copyright with the following copyright statement: Copyright: © 2017 Sellbrandt I et al.

Data associated with the article are available under the terms of the Creative Commons Zero "No rights reserved" data waiver (CC0 1.0 Public domain dedication). 

Dataset 1: Demographics, perioperative observations, and response to questionnaire of the patients undergoing surgical scrub and covering pre and post-anaesthesia. doi, 10.5256/f1000research.11965.d166034 ^14^
