# Supplementary material for: The choice between surgical scrubbing and sterile covering before or after induction of anaesthesia: A prospective study
Source: F1000Res. 2017 Aug 9;6:1019. Originally published 2017 Jun 28. [Version 2] doi: 10.12688/f1000research.11965.2 (PMC5553078; doi:10.12688/f1000research.11965.2)
Supplement: Peer review history

# Peer review: Referee response for version 2

**DOI:** 10.5256/f1000research.13385.r24882

**Published:** 2017-08-10

**Authors:** Jakob Walldén

**Affiliations:** 1 Anaesthesia and Intensive Care, Umeå University, Sundsvall, Sweden

The authors have adressed the issues raised in the first version adequetely. The manuscript is now clear and of good quality.

I have read this submission. I believe that I have an appropriate level of expertise to confirm that it is of an acceptable scientific standard.

# Peer review: Referee response for version 1

**DOI:** 10.5256/f1000research.12935.r24416

**Published:** 2017-08-04

**Authors:** Adam Magos

**Affiliations:** 1 Royal Free Hospital, London, UK

This is a simple, small prospective RCT addressing the issue of scrubbing and prepping patients undergoing hernia repair ("anaesthetised" group) before or after ("awake" group) induction of general anaesthesia. The manuscript is clearly written and provide adequate details about methodology, including a power calculation. The results show that the only statistically significant advantage an "awake" approach was less time in recovery by 9 (48 v 39) minutes. None of the other variables were significantly different although there was a trend to less anaesthetic usage in the awake group associated with a 4 (64 v 60) minutes reduction in anaesthetic time.

 Whether or not there is a meaningful advantage of pre-anaesthetic scrubbing and prepping is not confirmed by this study, perhaps because it was underpowered, and this is reflected in the authors' conclusions.

 Nonetheless, this is an interesting study and one which may stimulate larger studies in different surgical areas. For this reason, publication would be worthwhile.

I have read this submission. I believe that I have an appropriate level of expertise to confirm that it is of an acceptable scientific standard.

# Peer review: Referee response for version 1

**DOI:** 10.5256/f1000research.12935.r23882

**Published:** 2017-07-17

**Authors:** Jakob Walldén

**Affiliations:** 1 Anaesthesia and Intensive Care, Umeå University, Sundsvall, Sweden

**General comments:**

 Interesting pragmatic study evaluating if surgical scrub should be done before or after induction of anesthesia. The perspective is both from the patients and nurses  point of view. The authors have found no major differences between the approaches and the major conclusion of the study is adequate.

 My major concerns regarding the manuscript is that the authors must be more consistent with the endpoints in the study, there is no clear line in the manuscript of the primary and secondary endpoints. The power analysis is done on the reduction of an undefined vasoactive drug and this is consistent with the hypothesis stated in the end of the introduction, but this outcome is not even mentioned in the abstract. 

 Please define the endpoint in the study clearly, and be consistent with these endpoint when presenting and discussing the results throughout the manuscript.

 Please correct tense throughout the manuscript and use past tense when describing the study. Please also proof-read so that grammar is correct throughout the manuscript, there are still quite a few grammatical errors.

**Specific comments:**

**Abstract/Results**:  Present the figures of the main results, not only the p-values.

**Abstract/Conclusion:  ** Is the statement "safe" supported"? No outcomes regarding safety.

**Introduction:**

 Relevant section. Punctuation instead of question-mark in in last sentence of introduction.

**Methods:**

- Please use correct name of the review board (Regional Ethical Review Board  in Gothenburg). State date for decision.
- Please state main outcome variables, primary and secondary.
- Duplicated sections?

 

*Patients received all medication and care in accordance to*

*routine procedures of the department, apart from the scrubbing*

*and sterile covering. Premedication was with paracetamol and*

*diclofenac.*

*All patients received care in accordance to routines of the*

*department, apart from the preoperative preparation, surgical scrub*

*and sterile covering awake or after induction of anaesthesia.*

  

- How was the procedure/routine for giving vasoactive drugs during anesthesia? First choice, second choice? Blood pressure cutpoints?
- Power analysis: Reduction in what drug?

 

**Results and Table 1:**

 Present the results in a structured order according to primary and secondary outcomes.

 The primary endpoint (vasoactive drugs) is a parameter that might needs to be presented more extensively. Three drugs are presented, and there is a small tendency that the awake group received more vasoactive drugs. Another dimension to explore the data is to present the number of patients that needed vasoactive drugs.

 Nurse rating: unclear what you mean with:   / … follows: 1, 6/10; 2, 8/10./   Are there one nurse rating missing?

**Discussion:**

 Main conclusions adequate in first paragraph.

 In second paragraph regarding theatre nurses, there are many statements without references. ( ..prefer patients being asleep… distressful for patients…. freezing sensations…discussion regarding that awake patients may be at an increased risk for surgical site infections… ). Please support the statements if possible.

 Discuss more extensively the differences between the group and possible impact on the results (i.e. age differences, anesthetic doses). 

 Discuss if the study was properly powered. Better to use other variable calculate power?

I have read this submission. I believe that I have an appropriate level of expertise to confirm that it is of an acceptable scientific standard, however I have significant reservations, as outlined above.

# Response

**Published:** 2017-07-27

**Authors:** Jan Jakobsson

**Affiliations:** Department of Physiology and Pharmacology, Karolinska Institutet, Sweden

Thank you for adequate and constructive comments;

My major concerns regarding the manuscript is that the authors must be more consistent with the endpoints in the study, there is no clear line in the manuscript of the primary and secondary endpoints. The power analysis is done on the reduction of an undefined vasoactive drug and this is consistent with the hypothesis stated in the end of the introduction, but this outcome is not even mentioned in the abstract. 

Please define the endpoint in the study clearly, and be consistent with these endpoints when presenting and discussing the results throughout the manuscript.

*The study was set-up to study differences between the two logistics, sterile washing and dressing before or after induction of anaesthesia. The power analysis was based on a pilot study looking at the amount of ephedrine needed, with MAP 60 and SAP 90 as cut-off values.*

*Secondary outcomes where patients and scrub nurses assessment, any signs of safety concerns, and last theatre turn time around and PACU stay duration. We have now clarified and amended the manuscript accordingly.*

Please correct tense throughout the manuscript and use past tense when describing the study. Please also proof-read so that grammar is correct throughout the manuscript, there are still quite a few grammatical errors.

*We have amended the language to past tense and further checked spelling and grammar*

**

**Specific comments:**

**Abstract/Results**:  Present the figures of the main results, not only the p-values.

*Added accordingly*

**Abstract/Conclusion:  ** Is the statement "safe" supported"? No outcomes regarding safety.

*Amended/clarified*

**Introduction:**

Relevant section. Punctuation instead of question-mark in in last sentence of introduction.

**Methods:**

- Please use correct name of the review board (Regional Ethical Review Board in Gothenburg). State date for decision. *Amended/added *
- Please state main outcome variables, primary and secondary. Clarified
- Duplicated sections?

 

*Patients received all medication and care in accordance to*

*routine procedures of the department, apart from the scrubbing*

*and sterile covering. Premedication was with paracetamol and*

*diclofenac.*

*All patients received care in accordance to routines of the*

*department, apart from the preoperative preparation, surgical scrub*

*and sterile covering awake or after induction of anaesthesia.*

*Corrected*

- How was the procedure/routine for giving vasoactive drugs during anesthesia? First choice, second choice? Blood pressure cutpoints?
- Power analysis: Reduction in what drug? Efedrin

 

**Results and Table 1:**

Present the results in a structured order according to primary and secondary outcomes.

The primary endpoint (vasoactive drugs) is a parameter that might needs to be presented more extensively. Three drugs are presented, and there is a small tendency that the awake group received more vasoactive drugs. Another dimension to explore the data is to present the number of patients that needed vasoactive drugs.

Nurse rating: unclear what you mean with:   / … follows: 1, 6/10; 2, 8/10./   Are there one nurse rating missing?

**Discussion:**

Main conclusions adequate in first paragraph.

In second paragraph regarding theatre nurses, there are many statements without references. ( ..prefer patients being asleep… distressful for patients…. freezing sensations…discussion regarding that awake patients may be at an increased risk for surgical site infections… ). Please support the statements if possible.

Discuss more extensively the differences between the group and possible impact on the results (i.e. age differences, anesthetic doses). 

*Further commented*
